# Histone demethylase lysine demethylase 5B in development and cancer

**DOI:** 10.18632/oncotarget.13858

**Published:** 2016-12-10

**Authors:** Mengjiao Han, Wenxia Xu, Pu Cheng, Hongchuan Jin, Xian Wang

**Affiliations:** ^1^ Department of Medical Oncology, Key Laboratory of Biotherapy in Zhejiang, Sir Runrun Shaw Hospital, Medical School of Zhejiang University, China; ^2^ Department of Surgical Oncology, Key Laboratory of Cancer Prevention & Intervention, National Ministry of Education, Provincial Key Laboratory of Molecular Biology in Medical Sciences, Second Affiliated Hospital Zhejiang University School of Medicine, Hangzhou, China

**Keywords:** histone methylation, demethylases, KDM5B

## Abstract

Histone methylation is one of the most important chromatin posttranslational modifications. It has a range of influences on nuclear functions including epigenetic inheritance, transcriptional regulation and the maintenance of genome integrity. Changes in histone methylation status take part in various physiological and pathological processes. KDM5B (lysine demethylase 5B, also called JARID1B or PLU-1) encodes the histone H3 lysine4 (H3K4) demethylase and exhibits a strong transcriptional repression activity. KDM5B plays a role in cell differentiation, stem cell self-renewal and other developmental progresses. Recent studies showed that KDM5B expression was increased in breast, bladder, lung, prostate and many other tumors and promotes tumor initiation, invasion and metastasis. Given its association with tumor progression and prognosis of cancer patients, KDM5B was proposed to be a novel target for the prevention and treatment of human cancers. In this review, we will summarize recent advances in our understanding of the regulation and function of KDM5B in development and cancer.

## INTRODUCTION

Through the modulation of chromatin structure, histone post-translational modifications including methylation, acetylation, phosphorylation and ubiquitination play a significant role in creating transcriptional activation or repression patterns [1]. It is crucial for precise coordination and organization of the open and closed chromatin structure during many dynamic processes such as DNA replication, repair, recombination, transcription and so on. Changes in local or global chromatin structure have been found to be the key features of many if not all tumors, indicating that such epigenetic changes may make a potential contribution to carcinogenesis [2, 3].

Recent studies have demonstrated that histone methylation has profound effects on nuclear functions such as transcriptional regulation, maintenance of genome integrity and epigenetic inheritance [4]. For example, histone methylation on arginine or lysine residues can either activate or repress gene transcription, depending on which particular arginine or lysine residue becomes modified [5]. Methylation and demethylation on arginine or lysine residues in histone tails are reversible modifications that are tightly controlled by histone methyltransferases and histone demethylases [6]. Such dynamic balance of methylation and demathylation is frequently altered in tumorigenesis and pathogenesis of other disorders [6–9].

There are three histone methylation states: monomethyl (me1), dimethyl (me2) or trimethyl (me3) [10]. H3K4me1 is related with enhancer functions and participates in gene repression in metazoans [11, 12], nucleosome dynamics and chromatin regulation of yeast stress-responsive genes [13]. H3K4me2 is connected to gene repression and transcription in yeast [14, 15], whereas H3K4me3 is linked to active transcription and is present around transcriptional start sites [16, 17].

Lysine-specific demethylation is facilitated by two families of enzymes, of which the JmjC(JumonjiC) domain-containing family of histone demethylases (JHDMs) is the major one [5]. KDM5B (lysine-specific demethylase 5B), also known as JARID1B (jumonji AT-rich interactive domain 1B) or PLU-1, is one member of the JHDMs subfamily which has recently attracted much attention.

## STRUCTURE OF KDM5B

**Figure 1:**
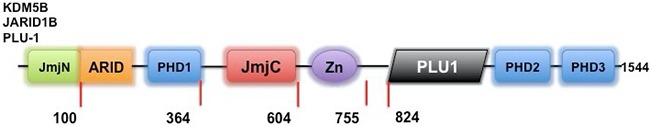
The structure of KDM5B JmjN: N-terminal Jumonji domain; ARID: AT-rich interactive DNA-binding domain; JmjC: Jumonji C-domain; Zn: C5HC2-type zinc finger domain; PHD: Plant homeodomain finger domains.

A comparison of the predicted amino acid sequence of the KDM5B across species reveals prominent homologies from plants to yeast, flies, worms and mammals [18]. Human KDM5B encodes a 1,544 amino acid protein with multi-domains [5]. KDM5B belongs to the KDM5 subfamily and also the JARID1 family, members of which contain an evolutionary conserved jumonji C-domain(JmjC). In general, JmjC demethylases can target mono-, di- and tri-methylated histone lysine residues such as lysine 4 on histone H3 [19]. JmjC-domain-containing histone demethylases catalyze demethylation of methylated histone lysines through an oxidative hydroxylation reaction mechanism that requires iron and a-ketoglutarate (a-KG) as cofactors and most likely happens when the affected methyl group is direct hydroxylation [20]. In addition to the catalytic JmjC domain, KDM5B also contains a N-terminal jumonji domain (JmjN), an ARID DNA-binding motif, a PLU1 motif, two or three methyl–lysine or methyl–arginine plant homeodomain (PHD) finger domains (PHD1, PHD2 and PHD3), and a C5CH2-type zinc finger domain (C-terminal helical zinc-binding domain) [21] (Figure 1). Each member performs a distinct function during various biological processes via binding to different chromosomal domains and participating in distinct enzymatic activities. The ARID DNA-binding motif is an AT-rich interactive domain that selectively binds DNA with CG-rich sequences [21]. A recent study focus on characterization of a linked Jumonji domain indicates that the ARID and PHD1 domains have little impact on the catalytic activity of the KDM5B whereas the linked JmjN-JmjC domains together with the immediate C-terminal helical zinc-binding domain are necessary for its enzyme catalytic activity [22]. Another study showed that PHD1 help KDM5B target downstream genes to mediate demethylation and exert tumor-suppressor functions [23]. The nuclear localization of KDM5B has been confirmed by using an antiserum that specifically recognizes the C-terminal end of KDM5B [24]. However, whether KDM5B can localize in the cytoplasm for demethylating cytoplasmic proteins remains controversial.

## REGULATION OF KDM5B

### Transcriptional regulation

The mouse Kdm5b shares 94% overall identity at the amino acid level with the human KDM5B. Like human KDM5B, the expression of Kdm5b is also restricted, with high expression observed only in testis and mammary gland. During the developing embryo, expression of Kdm5b is specifically limited to brain, whisker follicle, eye, teeth, thymus and so on, in a temporally constrained fashion.

KDM5B was initially identified as a gene that was up-regulated by the tyrosine kinase HER2 in human breast cancer cells and its expression was shown to be closely associated with the malignant phenotype in breast cancer [18, 25]. Similarly, its expression in normal adult tissues was detected only in the testis, ovary and transiently in the mammary gland of pregnant females [26]. On the basis of its high levels of expression in the testis and its specific relationship with cancer, Bente Madsen et al assumed KDM5B belongs to the family of testis-cancer antigens [25]. Together, the consistency between the human and mouse KDM5B/Kdm5b genes in both structure and expression pattern shows a high conservation of KDM5B function [27].

### Post-translational regulation

SUMOylation is a post-translational modification with important effects on histone lysine demethylases. KDM5B was reported to be SUMOylated at evolutionarily conserved lysine residues 242 and 278 located between the ARID and PHD domains. The SUMO E3 ligase human polycomb protein 2 (HPC2) was responsible for SUMOylation of KDM5B [28]. SUMOylation of KDM5B impaired its function by blocking its interaction with target genes. In addition, SUMOylated KDM5B was susceptible to RNF4-dependent ubiquitination subsequent proteasomal degradation [29].

Protein ubiquitination is one of the important post-translational modifications in eukaryotic cells [30]. KDM5B was regulated by ubiquitination as well. For example, S-phase kinase associated protein-2 (SKP2) was shown to increase the level of H3K4me3 through decreasing the K63-linked ubiquitination of KDM5B by E3 ubiquitin ligase TRAF6 in prostate cancer cells [31].

Phosphorylation also plays an important part in regulation of KDM5B relevant progression. Kdm5b depletion up-regulated extracellular Reln(reelin) concentration in neural stem cells(NSCs) from adult subventricular zone (SVZ) of mice and increased phosphorylation of the downstream reelin signaling target Disabled-1 (Dab1) [32]. It revealed that Kdm5b enriched at proximal promoter regions of Reln and negatively regulated neurogenesis [33]. Consistent with these findings, KDM5B depletion resulted in the loss of epithelial differentiation and increased the phosphorylation of c-Jun N-terminal kinase (Jnk/Sapk), which served as an inducer of senescence in colorectal cancer cells [34, 35]. In highly metastatic melanomas, re-expression of KDM5B can modulate retinoblastoma protein (pRb)-hypophosphorylation selectively at Ser795 to reveal tumor-suppressive function [36].

Accordingly, modification of the histone post-translation status due to changes in KDM5B activity may have profound effects on the gene transcription regulation and other nuclear processes. Next, we will discuss the potential function of KDM5B and its role in the tumor growth, development and progression.

## FUNCTION OF KDM5B

### Functions dependent on demethylase enzyme activity

Trimethylation of lysine 4 at histone 3 (H3K4me3) at transcription start sites actives transcription [37]. Genome-wide analyses of KDM5B occupancy have revealed its association with transcription start sites and its function in transcription repression by removing promoter-associated H3K4me3 from many target genes related to cell cycle, mitosis, proliferation, embryonic stem cell self-renewal, differentiation and other developmental aspects [37, 38]. KDM5B affected meiotic transcription in a manner that may be restricted to certain meiotic stages and may be mediated by the ability of this protein to associate with the chromatin [25]. KDM5B has been identified to be involved in the regulation of G2/M checkpoint and late M phase and in regulating the expression of a specific collection of genes involved in spindle assembly, chromosomal condensation, and transition through the late stages of mitosis [26]. In addition, KDM5B reduced the terminally differentiated cells and increased proliferating progenitors by facilitating G1/S transition [39]. KDM5B was believed to suppress downstream target genes expression such as the metallothionein (MT) genes by binding to the GCACA/C consensus sequence through its ARID domain [26]. KDM5B was also shown function in a repressive complex via Rb-mediated recruitment of KDM5B to remove the H3K4 activation mark from E2F-target genes in mouse and primary human cells and thus contributed their stable silencing during senescence [40, 41].

It was confirmed that KDM5B could target genes involved in the regulation of development and deciding cell fate. Embryonic stem cells (ESCs) constantly balance their potential to self-renew and differentiation [42]. One of the previous researches demonstrated that KDM5B was not a key molecule for ESCs self-renewal, but was essential for neural differentiation of ESCs. KDM5B was shown to bind to transcription start sites of genes encoding developmental modulators, and its inactivation led to increased levels of H3K4me3 at target promoters as well as globally [37]. While another study had described the essential role of KDM5B in ESCs self-renewal as a downstream Nanog target that was recruited to H3K36me3 through connecting to the chromodomain protein MRG15. KDM5B repressed cryptic initiation and maintained an H3K4me3 gradient that contributed to the regulation of transcription elongation [38]. By analysing the wide-range of genome occupancy of KDM5B in ESCs, KDM5B was concentrated at H3K4 methylation marks at both enhancers and promoters of active genes and regulated ESCs pluripotency by stopping them from spreading to gene bodied and enhancer shores [43]. Moreover, along the ESCs neural lineage, KDM5B was needed for the differentiation and its deficiency was related to the silencing of stem and germ cell genes [37]. Besides, KDM5B presented an obstacle to the reprogramming process and the epithelial-to-mesenchymal transition (EMT), which was downregulated during reprogramming when KDM5B was insufficient by expression analysis [44].

In addition to these functions, KDM5B might regulate genes required for DNA repair. PARylation of KDM5B mediated by PARP-1 was shown to inhibit the demethylation of H3K4me3 and its association with gene promoters [45]. There is growing evidence from embryonic and hematopoietic stem cells (HSCs) that KDM5B was a central regulator of key developmental genes [37, 46–48]. KDM5B was found to reduce the H3K4me3 levels and maintain the endothelial angiogenic capacity by exclusively occupying the promoter region of HOXA5 (an antiangiogenic factor) in a demethylase-dependent manner [49]. Results of an RNAi-based functional screen demonstrated that KDM5B functioned as a negative regulator of HSC activity [50]. While another research using formal genetics strongly suggested that KDM5B was a positive regulator of HSC self-renewal [51]. It was consistent with studies showing the importance of epigenetic regulation to HSC potential [52]. Therefore, whether KDM5B plays a positive or negative role in human hematopoiesis requires further functional validation.

### Function independent of demethylase enzyme activity

To determine possible molecular functions for KDM5B, a yeast two-hybrid screen using KDM5B as the bait was performed and identified two human transcription factors that interact with KDM5B, namely brain factor-1 (BF-1, also known as FOXG1b) and paired box 9 (PAX9) [53]. Since BF1 and PAX9 proteins interact with members of the Groucho co-repressor family, KDM5B might act as a specific transcriptional co-repressor of BF-1 and PAX9 via Groucho-mediated transcriptional repression. The KDM5B--BF-1--PAX9 interaction may play a significant role in embryogenesis as well as in breast cancer, but the mechanism is not very clear and further evaluation is required in the future [24]. In addition, KDM5B was reported to interact directly with other transcription repressors such as histone deacetylases (HDACs; both class I and class IIa) and indirectly with nuclear receptor corepressor (N-CoR). It also bound to the N-terminal half of HDAC4 containing the myocyte enhancer factor-2 (MEF2)-binding domain and this interaction seemed to depend on two functional PHD domains of KDM5B. KDM5B and HDAC4 were co-expressed during pregnancy as well as murine mammary glands involution, suggesting that it was possibly to have physiologically relevant between them [54]. KDM5B cooperated with polycomb repressive complex 2 (PRC2), directly interacted with its SUZ12 component and acting as a co-activator, rather than a co-repressor to stimulate Retinoic acid (RA)-induced gene transcription programs. This function depended on PRC2 enzymatic activity. The association between the JMJC domain of KDM5B and retinoic acid receptor α (RARα) was important for RA-mediated signaling pathway [55].

## RELEVANCE OF KDM5B TO HUMAN CANCERS

### KDM5B and breast cancer

KDM5B was initially identified as a gene that was up-regulated by the tyrosine kinase HER2 signaling in breast cancer and inhibited signaling from the c-erbB2 receptor. The expression of KDM5B was critical not only for key genes involved in mammary gland growth, but also for genes involved in breast carcinogenesis [56]. Increased expression of KDM5B in breast cancer was related with poor prognosis, suggesting that KDM5B had tumor promoting activities [18, 24]. A subsequent study verified an increased expression of KDM5B in human primary breast cancer samples compared with normal tissue and human breast cancer-derived cell lines [24]. Of all human cancer cell lines examined, the estrogen receptor (ER)-positive MCF-7 cell line showed the highest expression. Meanwhile, KDM5B knockdown increased G1 phase of MCF7 cells and retarded *in vitro* growth, colony formation in soft-agar and *in vivo* tumor formation [1, 57]. In addition, down-regulation of KDM5B by shRNA decreased tumor formation potential both in a syngeneic mouse mammary tumor model [1] and in xenografts models [58]. Knockdown of KDM5B led to up-regulation of tumor suppressor genes including BRCA1, CAV1, and HOXA5 [1] and resulted in an increased level of H3K4me3 at the chromatin region of these target genes [59]. Besides, KDM5B could epigenetically repress the expression of tumor suppressive let-7e [60]. Immunohistochemical detectiondemonstrated that protein expression of p16 and KDM5B was negatively correlated in invasive ductal breast carcinoma [61]. Another study highlighted a key role of KDM5B in luminal cell-specific gene expression. Furthermore, ER+ breast tumors with high KDM5B activity were associated with worse clinical outcome and resistance to endocrine therapy, suggesting that therapeutic targeting of KDM5B might be a potential anticancer strategy [62]. In some highly metastatic types such as triple negative breast cancer (TNBC), KDM5B exhibited increased expression levels through its downstream target MALAT1, a long non-coding RNA (lncRNA), to increase tumor migration and invasion, leading to a poor survival in TNBC. A negative regulator microRNA called has-miR-448 was found to disrupt KDM5B-MALAT1 signaling axis and prevent TNBC progression [63]. However, in addition to serving as an oncogene, KDM5B had been shown to have tumor-suppressive activities. KDM5B was observed to repress the expression of CCL14 (an epithelial derived chemokine) and suppress angiogenesis and metastasis [64]. Consistent with its anti-oncogenic function, KDM5B was downregulated in ER− breast cancer cells and overexpression of KDM5B suppressed genes involved in cell proliferation, immune response, as well as angiogenesis and cell migration [65].

### KDM5B and melanoma

KDM5B had been implicated as a tumor suppressor in malignant melanoma as its expression level was downregulated and it inhibited cell proliferation in an Rb-dependent manner [36, 66, 67]. In contrast, KDM5B was also reported to have oncogenic functions to promote melanoma maintenance and metastatic progression in immunodeficient mice [62, 68]. Knockdown of KDM5B led to an exhaustion of tumorigenesis in series transplantation experiments, indicating that KDM5B regulated stem cell-like properties in melanoma cells in a dynamic fashion [68]. It was substantiated that high KDM5B expression correlated with lower survival. Interestingly, several reports described a slow-cycling subpopulation of human melanoma cells that expressed high levels of KDM5B and could give rise to highly proliferative progeny with reduced KDM5B expression [66, 68, 69]. Depletion of the KDM5B^high^ slow-cycling phenotype either by gene knockdown or targeting its bioenergetic metabolism made melanoma more sensitive for a remarkable and long-lasting therapeutic effect [69]. On the contrary, the enhanced expression of KDM5B may be an early event in human melanoma progression compared with benign nevi and may not be associated with melanoma invasiveness, indicating that KDM5B might not be an appropriate choice as a prognostic marker [70]. Therefore, KDM5B seemed to have a dual role during melanoma progression, initially exhibiting anti-proliferative activity but progressively becoming necessary for continuous tumor growth and maintenance [71].

### KDM5B in other cancers

In addition to breast cancer and melanoma, overexpression of KDM5B had also been described in bladder cancer and lung cancer. It seemed to be essential for the proliferation and survival of these cancers. Famous oncogenes such as E2F1 and E2F2 were downstream genes in the KDM5B pathway [72, 73]. Recently, KDM5B was found to stimulate non-small cell lung cancer cell proliferation and invasion by affecting p53 expression [74]. In gastric cancer, KDM5B acted as an oncogene by regulating Akt pathway to promote cell growth and metastasis [75]. KDM5B was also up-regulated in advanced and metastatic prostate cancers (PCa) and promoted the activation of androgen receptor target genes [8, 9]. Furthermore, a bioinformatics analysis uncovered a conserved microRNA-29a (miR-29a) target site in the 3-untranslated region (UTR) of KDM5B and the aberrant expression of miR-29a could inhibit proliferation and induce apoptosis by decreasing the expression of KDM5B in PCa cells [76]. Additionally, KDM5B protein stability was regulated by S-phase kinase associated protein-2 (SKP2), which elevated H3K4me3 level to facilitate prostate carcinogenesis by promoting ubiquitination-dependent degradation of KDM5B [31].

KDM5B was frequently up-regulated in hepatocellular carcinoma (HCC) specimens compared with its expression in adjacent non-tumor tissues. Its expression level correlated well with tumor size, TNM stage, Edmondson grade, and poor prognosis. Knockdown of KDM5B could remarkably suppress HCC cell proliferation partly via up-regulation of p15 and p27 [77]. Moreover, KDM5B expression played a critical role in chemo-resistance and stem cell-like phenotype of neuroblastoma cells as KDM5B-silenced cells gained a decreased tendency for tumor sphere formation and invasion, but became more susceptible towards cisplatin treatment [78]. Besides, silencing KDM5B significantly inhibited oral squamous cancer stem cell activity, its migration and invasion ability, potentiated the tumor-inhibitory effects for radiation therapy [79]. KDM5B overexpression could also predict proliferation properties in head and neck squamous cell carcinoma and KDM5B knockdown resulted in G1 arrest and early apoptosis by suppressing Bcl-2 family members [80]. Similar results were observed in esophageal squamous cell carcinoma [81, 82], colorectal cancer [35], epithelial ovarian cancer(EOC) [83], cervical cancer, renal cell carcinoma [72], diffuse large B-cell lymphoma [84] and other leukemic cells lines [85]. KDM5B was up-regulated in multiple typed of leukemia and related to aberrant cell proliferation. A newly published study demonstrated a strong increase in the expression of KDM5B in high-risk B-cell precursor acute lymphoblastic leukemia (B-ALL)compared with normal bone marrow. They found out the Ikaros DNA-binding zinc finger protein could mediate the repression of KDM5B in a manner depending on the activity of the histone deacetylase HDAC1, and this could be impaired by pro-oncogenic casein kinase 2 (CK2) [86].

Despite recent discoveries in the area about functions of KDM5B during various tumor progressions (Table 1), we still do not fully know the mechanisms. The expression of KDM5B was related well with tumor size, TNM stage, grade and other clinical indexes in some kinds of cancers, but some were not. In general, mutual mechanisms may exist that enable cancer cells to survive in rough conditions even tumors are heterogeneous functionally and genetically [69]. The revelation that KDM5B is an H3K4 demethylase indicated that active removal of H3K4 methylation may lead to cancer [1]. Since the KDM5B--BF-1--PAX9 interaction played an important role in embryogenesis, it was possible KDM5B exerted similar functions in cancer development as well [24]. Target gene identification studies declared a cell- type-specific pattern of regulated genes which responded to the changes of KDM5B's level either overexpression or exhaustion [59]. The underlying mechanism still await further studies despite of these probable suppositions.

**Table 1:** The roles of KDM5B in various cancers were studied so far

Type of cancer	Functional classification	Specific functions	Ref.
Breast cancer	oncogenic/ tumor suppressive	First identified as an H3K4 demethylase, a specific DNA-binding protein and a potential downstream target of HER2/ERBB2. KDM5B promoted cell proliferation though transcriptional repressing tumor suppressor genes, luminal-high genes, specific microRNAs and contributed to poor survival. In some certain types of breast cancer like ER- or TNBC, KDM5B served as an anti-oncogenic player.	1,18,24,56-65
Melanoma	oncogenic/ tumor suppressive	High KDM5B expression was correlated with lower survival. KDM5B exerted tumor suppressive functions through many ways. KDM5B may have a dual role over time, first anti-proliferative but long-term tumor maintaining. Inhibition of mitochondrial respiratory chain decreased the slow-cycling KDM5B high cells and overcame drug-resistant.	36,63,66-71
Bladder cancer	oncogenic	KDM5B depletion resulted in suppression of cell growth through co-regulation of the E2F/RB1 pathway. KDM5B repressed connexin 26's expression.	72,73
Lung cancer	oncogenic	KDM5B depletion resulted in suppression of cell growth through co-regulation of the E2F/RB1 pathway. KDM5B stimulated cell proliferation and invasion by affecting p53.	72,74
Gastric cancer	oncogenic	KDM5B promoted cell growth and metastasis by regulating Akt pathway.	75
Prostate cancer	oncogenic	KDM5B promoted androgen receptor target genes’ activation. It was negative regulated by miR-29a and its protein stability was modulated by SKP2.	8,9,31,76
Hepatocellular carcinoma	oncogenic	KDM5B expression was related well with tumor size, TNM stage, edmondson grade, and poor prognosis.	77
Neuroblastoma	oncogenic	KDM5B promoted tumor sphere formation and invasion and resisted cisplatin treatment.	78
Oral squamous cancer	oncogenic	Silencing KDM5B inhibited CSC activity, migration and invasion, potentiated the effect of radiation therapy.	79
Head and neck squamous cell carcinoma	oncogenic	KDM5B knockdown resulted in G1 arrest and early apoptosis by suppressing Bcl-2 family members.	80
Esophageal squamous cell carcinoma	oncogenic	KDM5B knockdown suppressed cancer cell growth, invasion, sphere formation and tumorigenicity. A correlation was observed between KDM5B nuclear expression level and histological grade.	81,82
Colorectal cancer	oncogenic	KDM5B depletion induced cellular senescence and suppressed cancer cell growth.	35
Epithelial ovarian cancer	oncogenic	High KDM5B expression was associated with low PFS, OS and chemotherapy resistance.	83
cervical cancer	oncogenic	High KDM5B expression was associated with cancer cell growth and tumorigenicity.	72
renal cell carcinoma	oncogenic	High KDM5B expression was associated with cancer cell growth and tumorigenicity.	72
Diffuse large B-cell lymphoma	oncogenic	High frequency of KDM5B CT gene expression appeared to be a good candidate for therapy.	84
Leukemia	oncogenic	Depletion of KDM5B resulted in losing cell viability and inducing apoptosis.	85

### Targeting KDM5B for cancer treatment

Accumulating evidence for a causal role of aberrant histone demethylation in human cancers has led to attempts to target this histone demethylases for anti-cancer treatment. However, lots of studies have focused on other KDMs and the development of KDM5B inhibitors remains in the early stages. 2,4-pyridinedicarboxylic acid (2,4-PDCA) was identified as an inhibitor that suppressed the catalytic core of KDM5B [5]. However, it was not specific to KDM5B because it can inhibit many other Fe(II)- and α-KG-dependent enzymes. Some known JmjC histone demethylase inhibitors were identified as KDM5B inhibitors in a high throughput screen of more than 15,000 small molecules. For example, 2-4(4-methylphenyl)-1,2-benzisothiazol-3(2H)-one(PBIT) could inhibit KDM5B activity up to 95%, with an IC50 value of approximately 3 uM. However, it seemed to be a pan-KDM5 inhibitor since it can also inhibit KDM5A/B/C [87]. In addition, its safety and *in vivo* efficacy had not been tested. Therefore, much research is still needed to discover effective inhibitors that target KDM5B for the prevention and treatment of human cancers.

## CONCLUSIONS AND PERSPECTIVES

**Figure 2:**
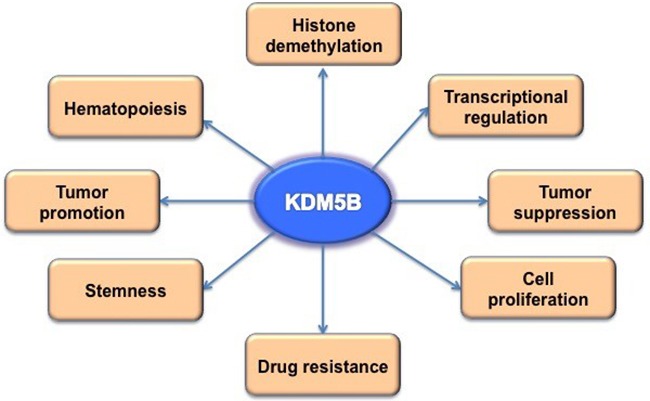
The biological function of KDM5B By regulating demethylation of histones and potentially other non-histone proteins, KDM5B is important in regulating gene transcription, cell differentiation and proliferation, as well as cancer progression including drug resistance and metastasis.

In conclusion, several lines of evidence suggest that the histone demethylase KDM5B plays an important role in the initiation and progression of many human cancers. Most of these functions are supposed to be related to its transcriptional repression activity by demethylation histones, although other non-histone proteins might also be regulated by KDM5B-dependent demethylation (Figure 2). The relevance of such unidentified targets of KDM5B needs to be clarified to facilitate the development of KDM5B inhibitors for anti-cancer treatment. Despite the successes of target therapy clinically, many of them target membrane or signaling molecules and it would be challenging to target KDM5B in the nucleus. In addition, the potency and specificity of KDM5B would be important due to the potential redundancy imposed by members of same family such as KDM5A and KDM5C. Nevertheless, any efforts to identify potential inhibitors of KDM5B will facilitate our understanding of its relevance to the development of human cancer and other disorders, eventually translating into KDM5B inhibitors for the clinical application.
